# Evaluation of cochlear implant electrode scalar position by 3 Tesla magnet resonance imaging

**DOI:** 10.1038/s41598-021-00824-3

**Published:** 2021-10-29

**Authors:** C. Riemann, L. U. Scholtz, H. B. Gehl, M. Schürmann, H. Sudhoff, I. Todt

**Affiliations:** 1 Department of Otolaryngology, Head and Neck Surgery, Medical School OWL, Bielefeld University, Campus Klinikum Bielefeld Mitte, Teutoburgerstr.50, 33604 Bielefeld, Germany; 2 Department of Radiology, Medical School OWL, Bielefeld University, Campus Klinikum Bielefeld Mitte, Bielefeld, Germany

**Keywords:** Diseases, Medical research

## Abstract

The estimation of scalar electrode position is a central point of quality control during the cochlear implant procedure. Ionic radiation is a disadvantage of commonly used radiologic estimation of electrode position. Recent developments in the field of cochlear implant magnets, implant receiver magnet position, and MRI sequence usage allow the postoperative evaluation of inner ear changes after cochlear implantation. The aim of the present study was to evaluate the position of lateral wall and modiolar cochlear implant electrodes using 3 T MRI scanning. In a prospective study, we evaluated 20 patients (10× Med-El Flex 28; 5× HFMS AB and 5× SlimJ AB) with a 3 T MRI and a T2 2D Drive MS sequence (voxel size: 0.3 × 0.3 × 0.9 mm) for the estimation of the intracochlear position of the cochlear implant electrode. In all cases, MRI allowed a determination of the electrode position in relation to the basilar membrane. This observation made the estimation of 19 scala tympani electrode positions and a single case of electrode translocation possible. 3 T MRI scanning allows the estimation of lateral wall and modiolar electrode intracochlear scalar positions.

## Introduction

Quality control in modern medicine plays an increasingly important role and follows structured evaluation pathways after an analysis of the contributing variables^1^. In otology, multiple methods and stages of quality control have been discussed and implemented^2^.

A central point in cochlear implantation quality control is based on the estimation of electrode inside the cochlea, originally performed with a 2D X-ray^3^. A further central point in the development and establishment of cochlear implant quality control was based on the work of Aschendorff et al.^4^, who provided a 3D impression of the electrode position for the first time by differentiating between scala tympani (ST) position, scala vestibuli (SV) position, and translocation between both scalae performed by flat panel tomography. The importance of this finding was underlined by the correlation of the electrode position and speech perception values showing significant correlation^5–7^. Based on these findings, quality control was not only a tool for the evaluation of surgeons’ abilities in the theater^8^, but a tool for the evaluation of different electrodes as well^9^.

The radiological results were confirmed by temporal bone findings^10^ and histological comparisons^11^. Related to this observations MSCT or DVT became part of the intraoperative or postoperative clinical routine for the evaluation of electrode position^12^.

The disadvantages of ionic radiation led to the evaluation of electrophysiological tools for the estimation of electrode translocations performed by a neural response telemetry (NRT) ratio^13^ with limitations of electrode design and manufacturer brand. EcochG was observed to give the surgeon information in terms of a translocation^14^.

Recently even a correlation between impedance measurements and translocation and even tip folding was observed^15,16^.

The technique of an overlay of the postoperative digital volume tomography/computed tomography (DVT/CT) and the preoperative magnet resonance imaging (MRI) integrated MRI for the first time into the evaluation of electrode position^17^, but reconstruction was time-consuming, and the use of postoperative ionic radiation persisted.

With the development of a new generation of cochlear implant magnets, knowledge about the importance of implant positioning of more than 8 cm away from the external auditory canal^18–20^, and intrascanning-head position^21^, a pain-free evaluation of the cochlea after cochlear implantation^22^ became possible without problems of magnetic artifacting.

Initial evaluations of MRI scanning showed the general possibility of estimating electrode translocation of perimodiolar electrodes^23^, but limitations to visual resolution persisted^24^. Model testing allowed the optimization of MRI sequence patterns to solve this problem^25^.

The aim of the present study is to show that an estimation of electrode translocation can be performed with different electrode designs using a 3 T MRI.

## Materials and methods

In this prospective study, 20 patients received cochlear implantation between March 2020 and January 2021. In all cases, the cochlear implant receiver magnet was positioned 8 cm or more behind the external auditory canal^19^. In addition to regular postoperative DVT for the estimation of electrode position, a 3 T MRI scanning was performed.

In this series, no inner ear anomalies, ossification, or tumors were included. In 10 cases, a Synchrony 1/2 Flex 28 (Med-El, Innsbruck, Austria) was implanted. In 5 cases, High Focus Midscalar (HFMS) 3D Advanced Bionics and SlimJ 3D Advanced Bionics (Advanced Bionics, Stäfa, Switzerland) devices were implanted. Individual data are shown in Table 1.

**Table 1:** Individual surgery date, electrode and estimated position by observer 1 and 2 for DVT/CT and MRI.

No	Surgery	Electrode	Observer 1 position DVT/CT	Observer 2 position DVT/CT	Observer 1 and 2 position MRI
20	5.3.20	Slim J	ST	ST	ST
19	4.5.20	Flex 28	ST	ST	ST
18	27.5.20	Flex 28	ST	ST	ST
17	13.8.20	Flex 28	ST	ST	ST
16	18.8.20	Flex 28	ST	ST	ST
15	22.9.20	Flex 28	ST	ST	ST
14	13.10.20	Flex 28	ST	ST	ST
13	15.10.20	HFMS	ST	ST	ST
12	29.10.20	HFMS	ST	ST	ST
11	6.11.20	HFMS	ST	ST	ST
10	15.11.20	HFMS	Translocation	ST	Translocation
9	17.11.20	Slim J	ST	ST	ST
8	26.11.20	HFMS	ST	ST	ST
7	1.12.20	Flex 28	ST	ST	ST
6	3.12.20	Flex 28	ST	ST	ST
5	15.12.20	Flex 28	ST	ST	ST
4	16.12.20	Slim J	ST	ST	ST
3	18.12.20	Slim J	ST	ST	ST
2	8.1.21	Flex 28	ST	ST	ST
1	28.1.21	Slim J	ST	ST	ST

MRI: Achieva 3 T, Philips Medical System, Best, Netherlands.

Sequence: T2 2D Drive MS, voxel size 0.3 × 0.3 × 0.9 mm; FOV 150 × 150, TE 100 ms TR 3000, TSE tact 17, multi shot, flip angle 90°, refocus control 120, metric 512; NSA 5, foldover: AP.

Angulation of the MRI scan was directed in line with the basal turn.

DVT: New TOM VGI, Verona, Italy.

Parameters: FOV 15 × 15 cm, 10.48–20.52 mAS, KV 110, 360° followed by 2D and 3D reconstruction at an external workstation (NNT, main station).

The MSCT Toshiba Aquilion 80 protocol was: slice thickness 0.5 mm, KV 120, MA 200, rot.time 0.75.

Electrode position in terms of scalar location (DVT/CT and MRI) was evaluated independently by one surgeon and a neuroradiologist. DVT/CT and MRI were evaluated independently from each other.

Patients gave written informed consent. The study was approved by the institutional review board (IRB) (HNO-KliBi, 001/2020) and the ethical board of the University Münster (2019-135-f-S).

### Statement of ethics

The data used to support this study’s findings are available from the corresponding author upon request. The study was approved by the institutional review board of the Klinikum Bielefeld, Germany (IRB-klibi-HNO-2020/001) and the ethical board of the University Münster (2019-135-f-S). Patients gave written informed consent for the use of their clinical records in this study. The study was conducted in accordance with the World Medical Association Declaration of Helsinki.

## Results

Evaluation of cochlear implant electrode by 3 T MRI showed 2 visual indicators of electrode position in the axial plain. The 2 visual indicators are the diminishing of the T2 signal by the electrode and the identification of the basilar membrane.

**Figure 1:**
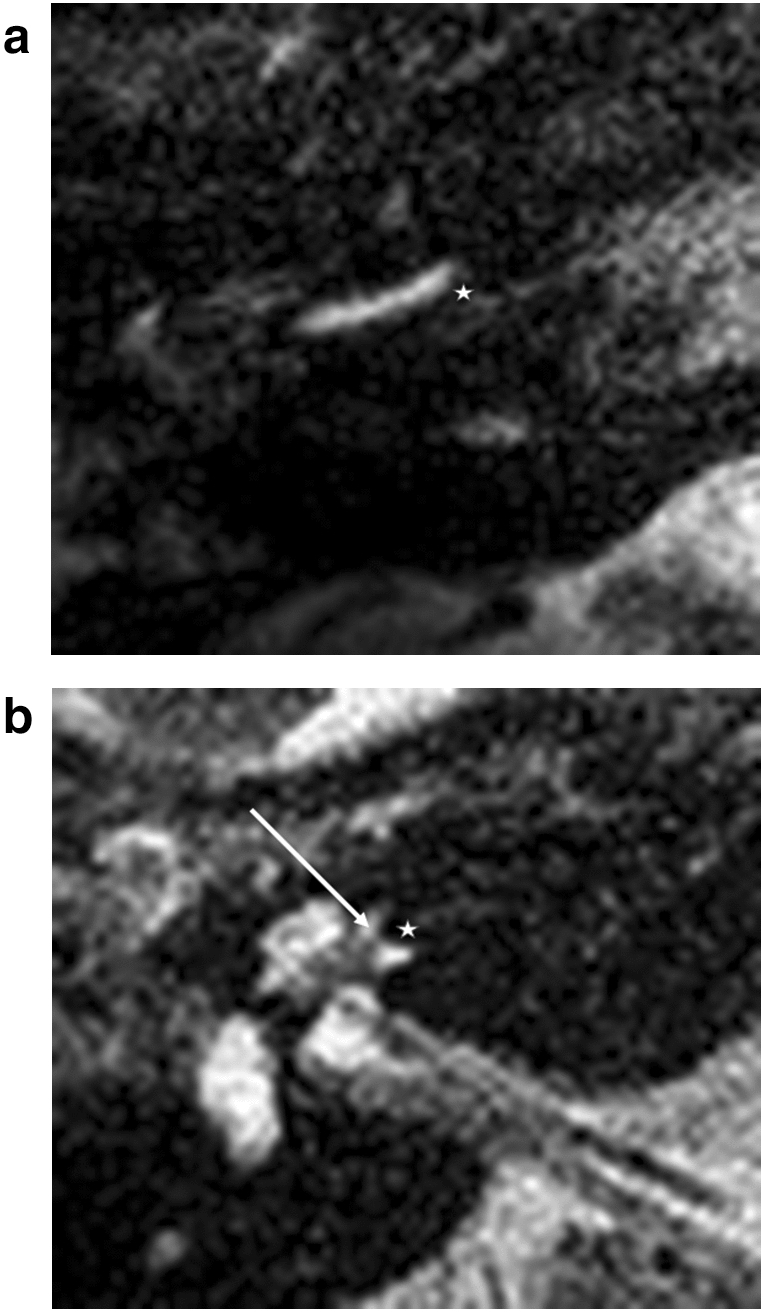
Straight electrodes, basal turn. (**a**) Flex 28, (**b**) SlimJ. Star represents diminishing electrode signal, arrow represents basilar membrane.

For the basal turn, a diminishing signal indicates the electrode localisation. At the lateral portion of the cochlea, each electrode was positioned depending on its design: either laterally, without any further lateral T2 signal (Fig. 1a,b), or more medially positioned with a T2 signal (Fig. 3). Lateral diminishing signal varied depending on electrode design: Flex 28 was round, and SlimJ laterally flatter. As a second indicator, a basilar membrane was visible. This basilar membrane diminishing signal allowed definitive estimation of the electrode location in the scala tympani or scala vestibuli.

**Figure 2:**
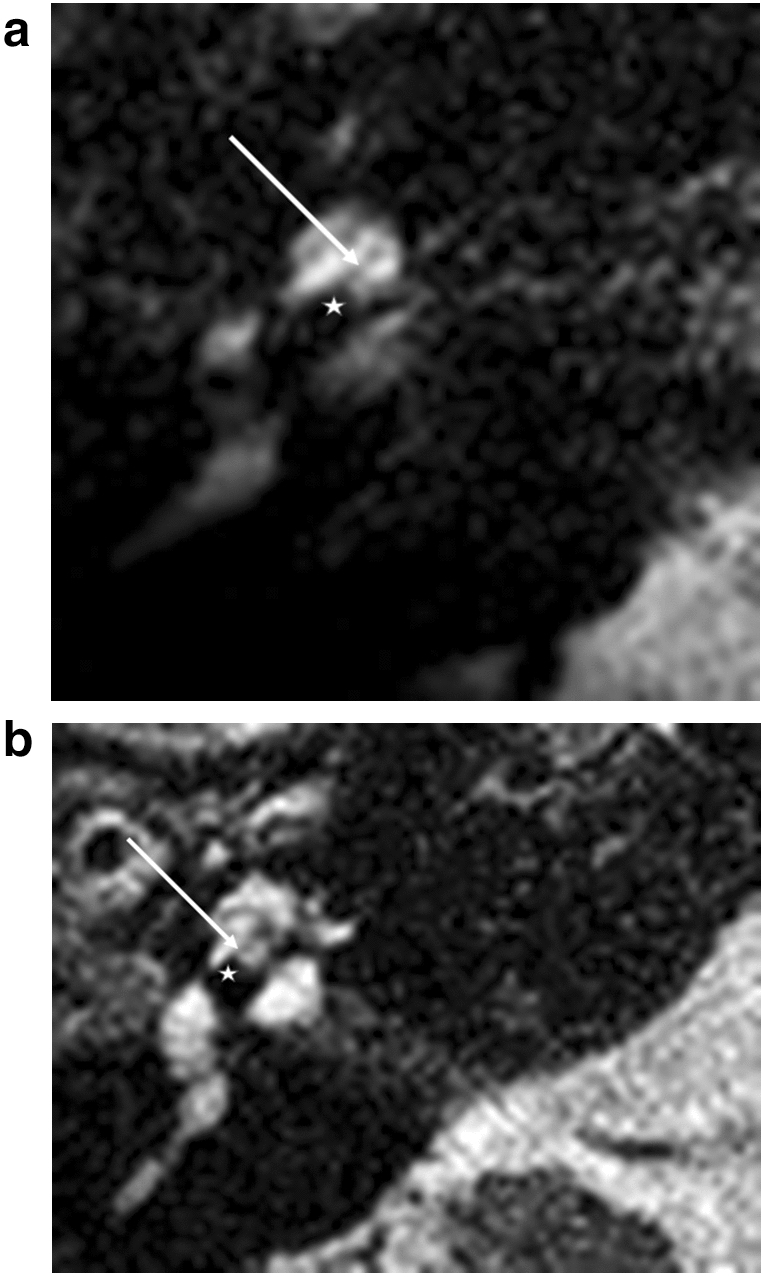
Straight electrodes, first turn. (**a**) Flex 28, (**b**) SlimJ. Star represents diminishing electrode signal, arrow represents basilar membrane.

At the first turn, a design-specific difference of the diminishing signal of the electrodes was obvious. We were able to differentiate between a round signal diminishing and a flatter signal diminishing (Fig. 2a,b). The basilar membrane signal (Fig. 2a,b) and the localization of the fluid signal diminishing indicate scala tympani positions at the first turn for these two types of lateral wall electrodes.

**Figure 3:**
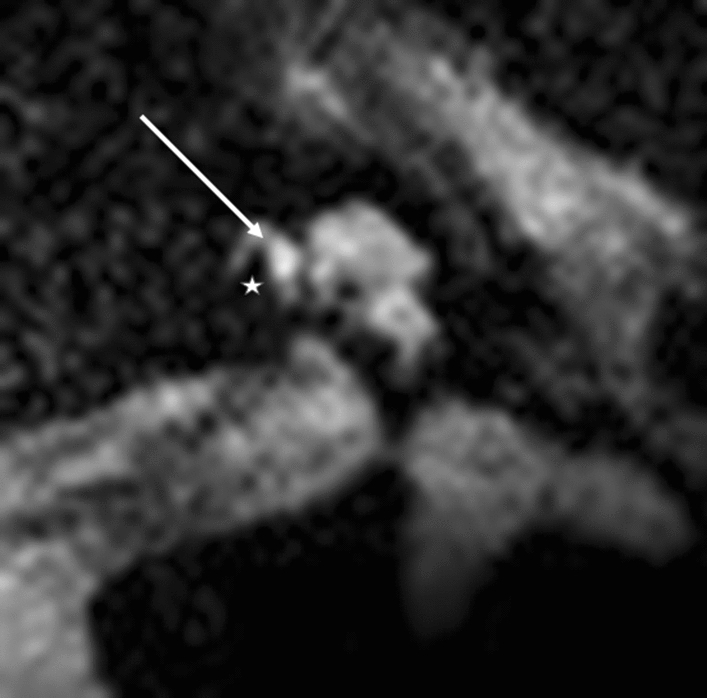
Modiolar electrode, basal turn, HFMS. Star represents diminishing electrode signal, arrow represents basilar membrane.

**Figure 4:**
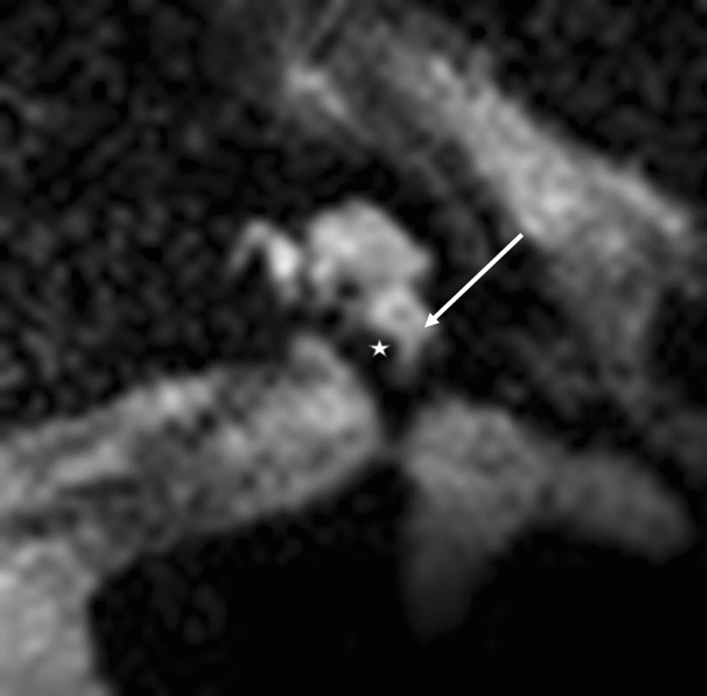
Modiolar electrode, first turn, HFMS ST Position. Star represents diminishing electrode signal, arrow represents basilar membrane.

Modiolar electrode characteristics allowed us to visualize a lateral T2 signal from the electrode at the basal turn and a basilar membrane pattern (Figs. 3 and 4). This basilar membrane pattern allowed us to locate the electrode in the scala tympani definitely.

**Figure 5:**
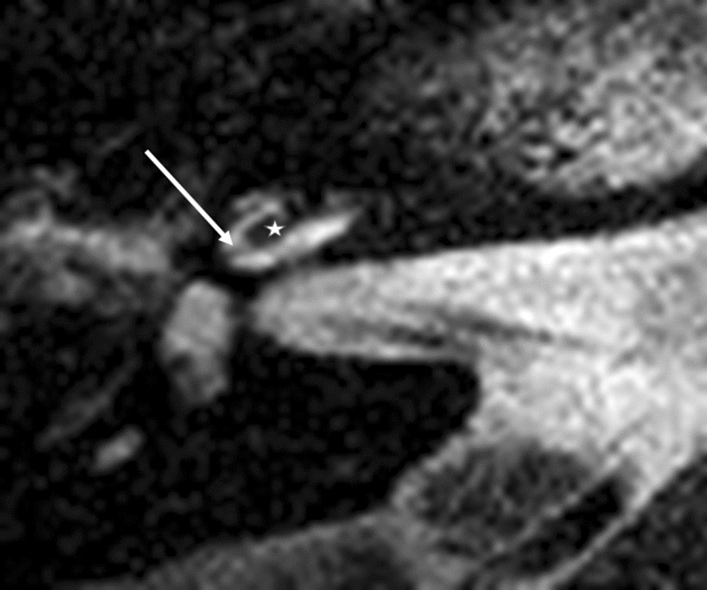
Modiolar electrode, first turn, HFMS SV Position. Star represents diminishing electrode signal, arrow represents basilar membrane. Pat.No.10.

**Figure 6:**
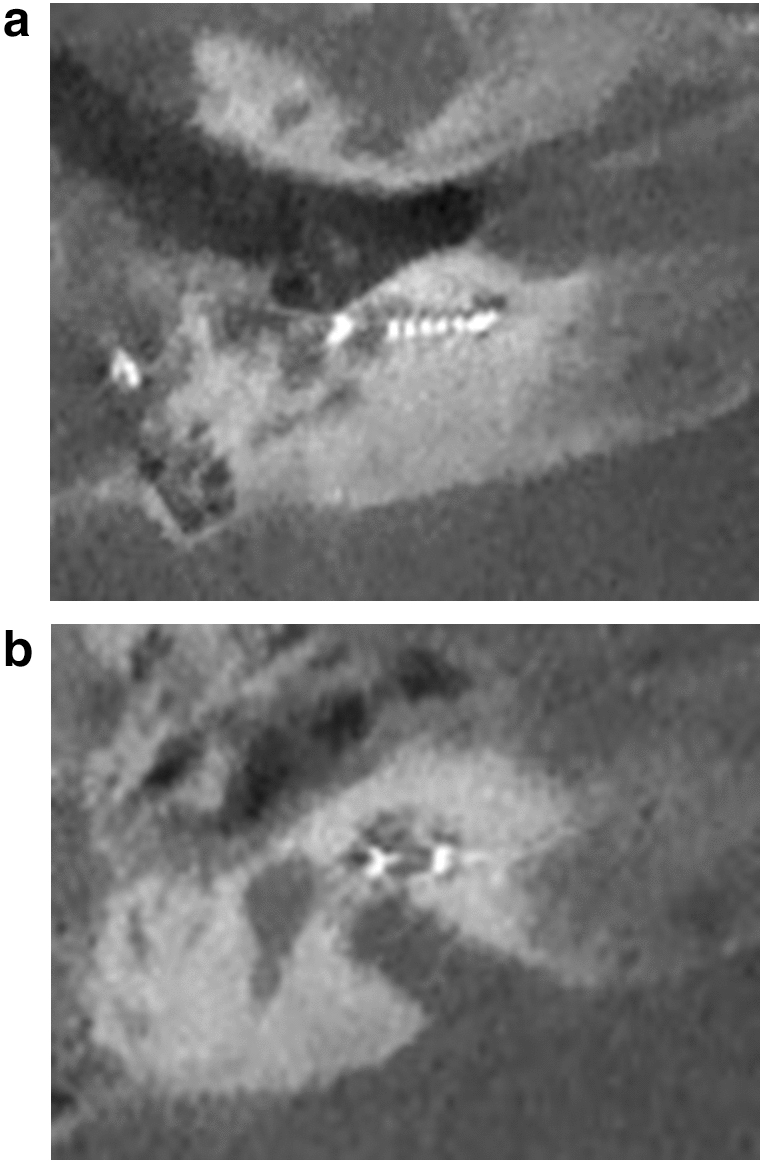
(**a**,**b**) Exemplary DVT estimated electrode position in the Scala tympani. (**a**) basal turn, (**b**) first turn at the floor of the cochlea.

**Figure 7:**
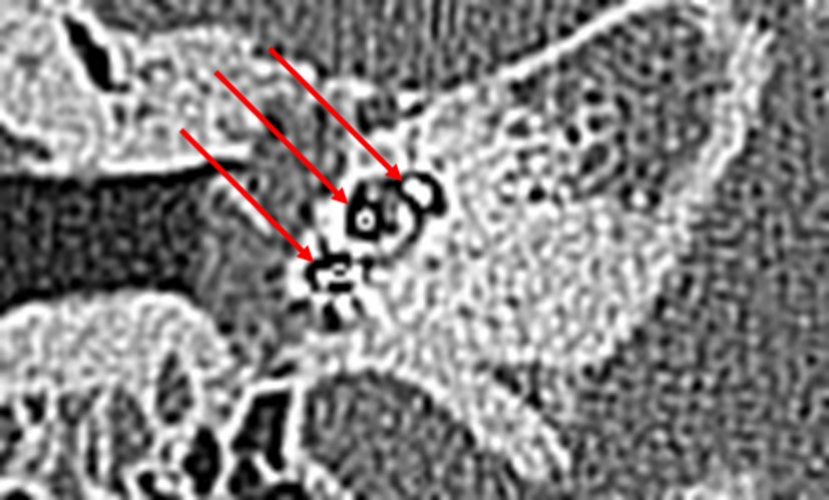
Translocated electrode. Arrows show electrode in the basal and first turn. MSCT of Pat. No.10.

At the first turn, the estimation of the electrode position depends on the estimation of the basilar membrane and the diminishing localisation of the T2 signal by the electrode. In Fig. 5, diminishing occurred above the T2 signal and the basilar membrane signal, indicating fluid. This indicated the pattern of an electrode translocated into the scala vestibuli. Figure 7 of the same patient (No.10) confirms the irregular high position of the electrode in the first turn. In Figs. 3 and 4, the signal diminished above the electrode and the basilar membrane, indicating an electrode position in the scala tympani. Figure 6a and b are exemplary DVT of regular electrode positions at the floor of the basal and the first turn.

Independent evaluation of electrode positions by one surgeon and a neuroradiologist using DVT/ CT and MRI confirmed the estimated MRI positions in all and in DVT/CT cases in almost all cases. In case 10 the estimation between the observer differed (Figs. 5 and 7, Table 1) (Test: Quadratic-Weighted Cohen’s κ with 95% confidence intervals; MRI: Kappa = 1.000; SE of kappa = 0.000, 95% confidence interval: From 0.704 to 1.00; DVT: Kappa = 0.898; SE of kappa = 0.099, 95% confidence interval: From 0.704 to 1.00).

Limitations of the study persist in terms of MRI resolution. Although voxel size of 0.3 mm could be used, a free reconstruction related to a non 3D sequence (building of iso voxel) was not possible. A 3 D MRI sequence commonly generates artifacts, which do not allow a visualization of the IAC and cochlea.

## Discussion

Quality control plays an important role in the structuration of medical pathways^1^. In otology quality control is well known and an important topic^2^. In the surgical portion of a cochlear implant procedure, electrode placement is of high importance for the audiological outcome^7^. The value of different radiological techniques for estimation of electrode positions lies in the evaluation of the surgeon ability of positioning and the properties of the electrode design to ensure the desired target position is met.

Different radiological techniques have been shown to determine electrode position in 2D^3^ or 3D^4^ patterns. The combination of postoperative DVT with an overlapping preoperative MRI allows electrode positions to be estimated relative to the MRI^17^.

The disadvantage of all these techniques is the occurrence of ionic radiation. Electrophysiological techniques such as the NRT ratio^13^, impedance^14^ and EcochG^15^ show promising results, but have limitations in terms of electrode design, are brand specific, depend on tissue properties or the intracochlear neural state.

Our technique demonstrates an option for estimating the electrode position without ionic radiation. This is clinically important, especially in children.

Our used MRI resolution [0.3 × 0.3 × 0.9 mm) differs substantially from high resolutions reached in temporal bones (µCT (0.06 mm isovoxel)] (MSCT (0.15 × 0.15 × 0.2 mm)^26^. But looking in the past, the difference in resolution between MRI, DVT and MSCT is under constant technical development.

This reached technical MRI refinement allows visual determination of the basilar membrane for the first time, underlining the important development of visual resolution in comparison to previous studies^23,24^. Our findings qualify the estimation of translocations for lateral wall electrodes and modiolar electrodes.

The usage of a commonly used 3 T MRI scanner underlines the option for regular usage of our technique in the clinical routine.

However, limitations persist for the visual estimation of insertion depths, anomalies, and specific electrode conditions such as tip folding. Costs of MRI in comparison to DVT or MSCT needs to be further discussed.

## Conclusion

3 T MRI scanning allows the estimation of lateral wall and modiolar electrode scalar positions.

## Data Availability

The datasets generated during and/or analysed during the current study are available from the corresponding author on reasonable request.
